# Expression level of dihydropyrimidine dehydrogenase is associated with clinical outcome in patients with T1G3 bladder cancer treated with Bacillus Calmette-Guerin

**DOI:** 10.1186/1756-0500-7-646

**Published:** 2014-09-13

**Authors:** Hiroki Ide, Eiji Kikuchi, Shuji Mikami, Akira Miyajima, Mototsugu Oya

**Affiliations:** Department of Urology and Pathology, Keio University School of Medicine, 35 Shinanomachi, Shinjuku-ku, Tokyo, 160-8582 Japan

**Keywords:** Urothelial carcinoma, S-1, 5-fluorouracil, Dihydropyrimidine dehydrogenase, Thymidylate synthase

## Abstract

**Background:**

Recently, it has been shown that 5-fluorouracil (5-FU) with a strong dihydropyrimidine dehydrogenase (DPD) inhibitor elicits a significant response in bladder cancer with a high level of DPD. However, only a few studies investigated the association between the level of the enzyme that regulates the metabolism of 5-FU and prognosis in bladder cancer. Furthermore, to our knowledge, there has also been no such report in T1G3 bladder tumors treated with BCG. Therefore, we evaluated enzymes that regulate the metabolism of 5-FU in T1G3 tumors treated with BCG immunotherapy using the Danenberg tumor profile (DTP) method, a highly accurate measurement of RNA from paraffin-embedded specimens.

**Methods:**

This study included 28 patients with T1G3 bladder cancer, each of whom underwent complete transurethral tumor resection and BCG intravesical instillation at our institution. The median follow-up period was 39 months (range, 3 to 159 months). The DTP method was used to analyze the mRNA expression of 3 enzymes related to 5-FU: DPD, orotate phosphoribosyltransferase (OPRT), and thymidylate synthase (TS).

**Results:**

Among the 28 patients, 13 developed recurrences (46.4%) and 5 experienced disease progression (17.9%). An elevated DPD mRNA level was significantly associated with recurrence (p = 0.048) and progression (p = 0.045). However, TS and OPRT mRNA levels were not significantly associated with any other clinical features or outcomes. Furthermore, the high DPD group had a significantly lower recurrence-free survival rate than the low DPD group (p = 0.047). Among patients with low DPD, the 2- and 5-year recurrence-free survival rates were 88.9% and 74.1%, respectively; while among patients with high DPD, the corresponding rates were 61.3% and 36.8%, respectively. TS and OPRT were not significantly associated with recurrence-free survival rates.

**Conclusion:**

DPD is significantly associated with recurrence and progression among T1G3 bladder cancer patients treated with BCG.

## Background

5-Fluorouracil (5-FU) has been used clinically to treat various cancers such as gastric, colon and bladder cancers. Previous studies have shown that enzymes that regulate the metabolism of 5-FU are significantly associated with the prognosis of bladder cancer, based on isotope labeling analyses [1–3]. Such enzymes include thymidylate synthase (TS), which is a target enzyme of 5-FU; dihydropyrimidine dehydrogenase (DPD), which degrades the majority of 5-FU; and orotate phosphoribosyltransferase (OPRT), which is the principal enzyme that converts 5-FU directly into an active antitumor metabolite. Furthermore, Mizutani et al. have demonstrated a significant association between enzymes that regulate the metabolism of 5-FU and tumor recurrence in cases of low-grade non-muscle-invasive bladder cancer [4]. Their report showed that patients with both low TS and high DPD activities had substantially higher rates of recurrence-free survival (RFS) than other patients. Accordingly, they suggested that a combination of TS and DPD activities might provide a significant prognostic indicator for low-grade non-muscle-invasive bladder cancer. Actually, uracil/tegafur (UFT), which is a 5-FU-based agent, has been shown to be effective in cases of low-grade non-muscle-invasive bladder cancer and S-1, a novel 5-FU-based agent with a strong DPD inhibitor, has a significant response compared to UFT in bladder cancer with a high level of DPD [5, 6]. However, to the best of our knowledge, no study has ever evaluated the association between enzymes that regulate the metabolism of 5-FU and cancer outcomes for cases of high-grade non-muscle-invasive bladder cancer.

In general, bladder cancer is clinically characterized by a high rate of recurrence and has a poor prognosis after tumors invade the muscle layer [7–10]. T1G3 bladder tumors have a particularly high risk of recurrence and progression to muscle invasion or metastatic disease. Intravesical Bacillus Calmette-Guerin (BCG) immunotherapy is a highly successful therapy for preventing tumor recurrence in cases with non-muscle invasive bladder cancer, but is not significantly associated with reduced disease progression [11, 12]. In practice, approximately 50% of T1G3 patients who receive BCG therapy ultimately develop recurrence, and half the patients who develop recurrence face disease progression that necessitates total cystectomy [13]. However, to our knowledge, few studies have investigated prognostic predictors for cases of T1G3 bladder tumors treated with BCG.

Danenberg et al. developed a methodology to evaluate gene expression in formalin-fixed paraffin embedded (FFPE) specimens [14]. This newly developed methodology has several advantages over previous methods, specifically providing more accuracy and practicality. There are various methods for measuring the levels of enzymes that regulate the metabolism of 5-FU. Among them, standard polymerase chain reaction (PCR), biochemical assays, and most other conventional techniques are not practical because these measurements usually require fresh samples. In fresh frozen samples of cancer, contamination with cancer stroma and even normal tissue cannot be avoided. Therefore, previous studies of enzymes that regulate the metabolism of 5-FU have relied on immunohistochemistry, which can be evaluated from FFPE specimens, but are less accurate and precise. Meanwhile, the Danenberg tumor profile (DTP) method provides improved accuracy because it involves measuring gene expression from FFPE specimens [15]. Furthermore, laser capture microdissection (LCM) provides selective isolation of a defined cell population from heterogenous tissue sections [16]. Thus, DTP with LCM is clinically relevant and can provide accurate information for predicting the outcomes of patients with cancer.

In the present study, using DTP with LCM, we evaluated the association of enzymes that regulate the metabolism of 5-FU (including DPD, TS, and OPRT) with the clinical characteristics and outcomes, with a special focus on T1G3 bladder cancer treated with BCG therapy.

## Methods

### Patient population

Surgical specimens from 45 patients who had been surgically treated for primary T1G3 bladder tumor with complete transurethral resection at Keio University Hospital from 1993 to 2006 were examined. Patients with an upper urinary tract tumor at the time of diagnosis or incomplete clinical data were excluded from the study. Seventeen patients who did not receive adjuvant BCG treatment were also excluded. The final study sample was composed of 28 patients with T1G3 tumors who had received BCG therapy. The regimen consisted of weekly BCG instillations at a dose of 80 mg for the Tokyo strain, or 81 mg for the Connaught strain, for 6–8 weeks.

Follow-up consisted of cystoscopy with cytology, which was performed every 3 months for the first 2 years, every 6 months for the next 3 years, and annually thereafter. Distant metastasis and upper urinary tract tumor recurrence were evaluated by performing intravenous urography, ultrasonography, or computed tomography scanning. Such evaluations were performed every 1 or 2 years for 5 years after the initial treatment. Recurrence was defined as a new tumor occurring in the bladder, and progression was defined as muscular invasion (stage T2 or higher) or metastatic disease.

The study itself was approved by the Keio University Ethics Committee. All specimens were fixed in 10% formalin and embedded in paraffin, and all slides were re-reviewed by an uropathologist. Tumors were staged according to the American Joint Committee on Cancer–Union Internationale Contre le Cancer TNM classification. Tumor grading was assessed according to the 1998 WHO/International Society of Urology Pathology consensus classification [17]. Lymphovascular invasion was defined as the presence of tumor cells within an endothelium-lined space without underlying muscular walls.

### Microdissection of primary tumors

Sections (10-μm thick) were obtained from areas identified as having the highest tumor concentration and then mounted on uncoated glass slides. For histology, representative sections were stained with hematoxylin and eosin according to the standard method. Before microdissection, sections were deparaffinized in xylene for 10 minutes and hydrated with ethanol solutions of 100%, 95%, and finally 70%. Sections were then washed in H_2_O for 30 seconds, stained with nuclear fast red (NFR, American MasterTech Scientific, Lodi, CA) for 20 seconds, and rinsed again in H_2_O for 30 seconds. Finally, the samples were dehydrated with ethanol solutions of 70%, 95%, and 100% for 30 seconds each, followed by xylene again for 10 minutes. The slides were then completely air-dried. The sections of interest were selectively isolated by using LCM (P.A.L.M. Microsystem, Leica, Wetzlar, Germany), according to the standard procedure [18]. Cancer cells and cancerous stroma of the sample were dissected by using the LCM technique. At least 25 mm^2^ of tumor tissue and stromal tissue were collected from each FFPE block.

### RNA Extraction and analysis of mRNA level

The dissected tissue samples were transferred to reaction tubes containing 400 μL of 4 M dithiothreitol (DTT)-GITC/sarc (4 M guanidinium isothiocyanate, 50 mM Tris–HCl, 25 mM EDTA) (Invitrogen; No. 15577–018). The blinded tissue samples (400 μL) designated for extraction were placed in a 0.5 mL thin-walled tube. The samples were homogenized and an additional 60 μL of GITC/sarc solution was added. They were heated at 92°C for 30 minutes and then transferred to a 2 mL centrifuge tube. Fifty μL of 2 M sodium acetate, pH 4.0, were added, followed by 600 μL of freshly prepared phenol/chloroform/isoamyl alcohol (250:50:1). The tubes were vortexed for 15 seconds, placed on ice for 15 minutes, and then centrifuged at 13,000 rpm for 8 minutes in a chilled (8°C) centrifuge. The upper aqueous phase was carefully removed and placed in a 1.5 mL centrifuge tube. Glycogen (10 μL) and 300–400 μL of isopropanol were added, and the samples were vortexed for 10–15 seconds. The tubes were chilled at −20°C for 30–45 minutes to precipitate the RNA. The samples were then centrifuged at 13,000 rpm for 7 minutes in an 8°C centrifuge. The supernatant was poured off and 500 μL of 75% ethanol was added. The tubes were again centrifuged at 13,000 rpm for 6 minutes in a chilled (8°C) centrifuge. The supernatant was then carefully poured off, so as not to disturb the RNA pellet, and the samples were quick-spun for another 15 seconds at 13,000 rpm. The remaining ethanol was removed and the samples were left to air-dry for 15 minutes. The pellet was resuspended in 50 μL of 5 mM Tris. Finally, the cDNA was prepared according to the method of Lord and colleagues [19].

Quantification of the 3 genes-of-interest (TS, DPD, and OPRT) as well as an internal reference gene (β-actin) was performed using a fluorescence-based real-time detection method (ABI PRISM 7900 Sequence detection System, TaqMan®, Perkin-Elmer (PE) Applied Biosystem, Foster City, CA). The PCR reaction mixture consisted of 1,200 nM of each primer, 200 nM of probe, 0.4 U of AmpliTaq gold polymerase, 200 nM each of dATP, dCTP, dGTP and dTTP, 3.5 mM of MgCl_2_ and 1 × Taqman buffer A containing a reference dye. The final volume of the reaction mixture was 20 μL (all reagents were obtained from PE Applied Biosystems, Foster City, CA). Cycling conditions were 50°C for 2 minutes, 95°C for 10 minutes, followed by 46 cycles of 95°C for 15 seconds and 60°C for 1 minute. The following primers and probe sequences were used: TS primers: GCCTCGGTGTGCCTTTCA and CCCGTGATGTGCGCAAT, probe 6FAM (carboxyfluorescein)-TCGCCAGCTACGCCCTGCTCA; DPD primers: AGGACGCAAGGAGGGTTTG and GTCCGCCGAGTCCTTACTGA, probe 6FAM-CAGTGCCTACAGTCTCGAGTCTGCCAGTG; OPRT primers: TAGTGTTTTGGAAACTGTTGAGGTT and CTTGCCTCCCTGCTCTCTGT, probe 6FAM-TGGCATCATTGACCTTCAAGCCCTCCT; β-actin primers: TGAGCGCGGCTACAGCTT and TCCTTAATGTCACGCACGATTT, probe 6FAM-ACCACCACGGCCGAGCGG. TaqMan® measurements yield Ct values that are inversely proportional to the amount of cDNA in the tube. For example, a higher Ct value means that more PCR cycles are required to reach a certain level of cDNA detection. Gene expression values (relative mRNA levels) are expressed as ratios (differences between the Ct values) between the gene of interest and an internal reference gene (β-actin). This reference gene provided the baseline measurement for the amount of RNA isolated from a specimen.

### Statistical analysis

All data are presented as the mean ± standard error (SE). Associations between enzymes that regulate the mechanism of 5-FU and clinicopathological features were assessed using the Mann–Whitney U-test or the chi-square test. RFS was estimated using the Kaplan–Meier method, and analyzed with the log-rank test. As described previously [20], continuous pretreatment clinical measurements were analyzed as dichotomous variables according to approximately “optimal” cutpoints as follows. The value that best discriminated between good and poor survival (i.e., which had the most significant *P* value in a log-rank test) was found by testing all possible cutpoints. All such cutpoints were then rounded to clinically relevant (i.e., convenient) values. The level of statistical significance was set at p < 0.05. These analyses were performed with the SPSS statistical software package, version 16.0 (SPSS: An IBM Company, Chicago, IL).

## Results

### Clinical characteristics in overall patients

The median duration of follow-up was 39 months (range, 3 to 159 months), and median patient age was 71 years (range, 34 to 83 years). Of the 28 patients treated with BCG, 13 developed recurrence (46.4%) and 5 experienced disease progression (17.9%). Of the 28 patients treated with BCG, 1 died of a cause other than bladder cancer, and no patient died of bladder cancer (Table 1).

**Table 1:** **Clinical characteristics in T1G3 patients treated with BCG**

	Overall
Age, mean ± SE	66.9 ± 2.3
Gender, men/women	23 (82.1%)/5 (17.9%)
Concomitant CIS	7 (25.0%)
Multiple disease	19 (67.9%)
LVI positive	18 (64.3%)
Recurrence	13 (46.4%)
Progression	5 (17.9%)
Death	1 (2.2%)
Disease specific death	0 (0.0%)

*SE*: standard error; *CIS*: carcinoma in situ; *LVI*: lymphovascular invasion.

### Associations between the clinical variables and enzymes that regulate the metabolism of 5-FU

The mean ± SE mRNA levels of TS, DPD, and OPRT in overall T1G3 samples analyzed (n = 28) were 9.40 ± 1.62, 1.21 ± 0.26, and 1.99 ± 0.21, respectively. There were no significant differences between any of the clinical variables and levels of enzymes that regulate the metabolism of 5-FU (Table 2).

**Table 2:** **The association between clinical variables and 5-FU related enzymes in T1G3 patients treated with BCG**

		TS (mean ± SE)	DPD (mean ± SE)	OPRT (mean ± SE)
Age				
Less than 70 (n = 14)		10.58 ± 2.12	1.02 ± 0.23	1.91 ± 0.21
70 or greater (n = 14)		8.23 ± 1.01	1.44 ± 0.26	2.08 ± 0.23
p value		0.664	0.268	0.964
Gender				
Men	(n = 23)	9.55 ± 1.34	1.35 ± 0.21	1.88 ± 0.15
Female	(n = 5)	8.16 ± 1.21	0.87 ± 0.34	2.18 ± 0.44
p value		0.748	0.123	0.748
Concomitant CIS				
Yes	(n = 7)	10.80 ± 2.23	0.64 ± 0.21	2.58 ± 0.37
No	(n = 21)	8.94 ± 1.25	1.31 ± 0.21	1.95 ± 0.15
p value		0.341	0.057	0.054
Multiple disease				
Yes	(n = 19)	8.98 ± 1.22	1.57 ± 0.38	1.77 ± 0.17
No	(n = 9)	10.05 ± 2.26	1.12 ± 0.18	2.26 ± 0.29
p value		0.770	0.534	0.130
LVI				
Yes	(n = 18)	9.64 ± 1.33	1.03 ± 0.19	1.91 ± 0.19
No	(n = 10)	8.72 ± 1.93	1.64 ± 0.32	2.09 ± 0.23
p value		0.547	0.055	0.817

*SE*: standard error; *CIS*: carcinoma in situ; *LVI*: lymphovascular invasion; *TS*: thymidylate synthase; *DPD*: dihydropyrimidine dehydrogenase; *OPRT*: orotate phosphoribosyl transferase.

### Associations between clinical outcomes and enzymes that regulate the metabolism of 5-FU

**Figure 1:**
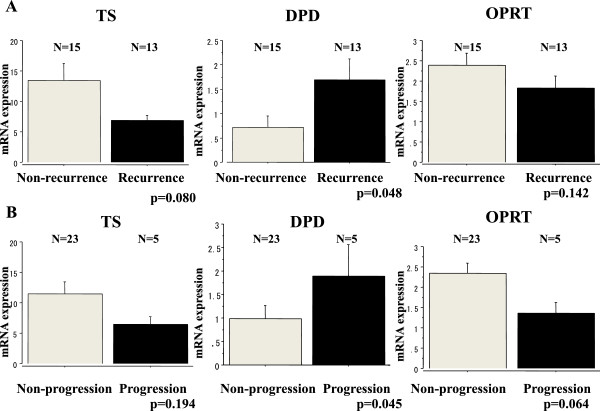
**Comparison of 5-FU related enzyme mRNA expressions between patients with and without recurrence/progression. (A)** The relative TS DPD and OPRT mRNA expressions between patients with and without recurrence; bars, +SE. **(B)** The relative TS DPD and OPRT mRNA expressions between patients with and without progression; bars, +SE.

Tumor recurrence was observed in 13 patients (46%) during follow-up. In overall T1G3 samples, the mean ± SE mRNA level of DPD was 1.69 ± 0.42 in the recurrence group, which was significantly higher than the mean level in the non-recurrence group (0.72 ± 0.23) (Figure 1A).Stage progression was observed in 5 patients (18%). The mean ± SE mRNA levels of DPD were 1.88 ± 0.68 in the progression group, which was significantly higher than the mean level in the non-progression group (0.98 ± 0.28) (Figure 1B). However, TS and OPRT levels were not significantly associated with recurrence or progression (Figure 1A and B).

**Figure 2:**
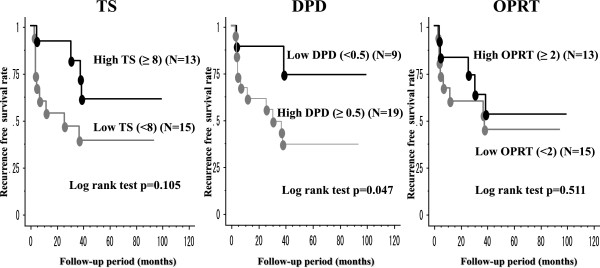
**Kaplan-Meier curves of recurrence-free survival of the patients with high and low 5-FU related enzyme expressions.** Kaplan-Meier curves of recurrence-free survival according to TS, DPD and OPRT expressions; bars, ± SE.

When DPD levels were discretized into two groups (as described in our Methods and previously [20]), the high DPD group (DPD ≥ 0.5) had a significantly lower RFS rate than the low DPD group (DPD < 0.5) (p = 0.047) (Figure 2). Furthermore, the low DPD group had 2- and 5-year RFS rates of 88.9% and 74.1%, respectively, while the high DPD group had corresponding RFS rates of 61.3% and 36.8%. We found no significant relationship between RFS rates and TS or OPRT (Figure 2).

## Discussion

Approximately 50% of patients who have T1G3 bladder cancer and receive BCG therapy survive without recurrence. On the other hand, half of the patients who develop recurrence also experience disease progression to muscle invasion [13]. Therefore, some cases of T1G3 bladder cancer have a malignant potential that cannot be prevented by using BCG therapy. However, no marker has been identified that can reliably predict the outcome of patients with T1G3 who receive BCG therapy. To our knowledge, only a few studies have evaluated significant predictors of clinical outcomes for T1G3 bladder cancers among patients treated with BCG. Lebret et al. have demonstrated that p53 is not a viable indicator of recurrence among patients with T1G3 bladder cancer [21]. Meanwhile, Alvarez-Múgica et al. found that high levels of myopodin methylation expression were significantly associated with increased rates of recurrence and progression among these patients, as well as shorter disease-specific survival times [22].

Previous studies found that enzymes that regulate the metabolism of 5-FU (such as TS, DPD, and OPRT) were significant prognostic factors for urothelial carcinoma (UC) of the bladder and upper urinary tract [1, 6, 23]. However, to our knowledge, no previous report has focused on patients who received BCG therapy for T1G3 bladder cancer. Furthermore, previous reports have measured the levels of enzymes that regulate the metabolism of 5-FU by using isotope labeling or immunohistochemistry, which provide less accurate measurements than the DTP method [15]. Therefore, we used the DTP method to evaluate associations between DPD levels and prognosis among patients who received BCG for T1G3 bladder cancer and found that DPD mRNA was a significant predictor of recurrence and progression among these patients.

In previous reports on patients with UC, DPD expression was not a significant prognostic factor [2, 6]. Mizutani et al. found that patients with high DPD had a lower RFS rate than patients in the low DPD group, although the difference was not statistically significant [2]. However, it has also been reported that DPD expression is significantly associated with tumor grade, perhaps suggesting that UC cells with high DPD expression have malignant potential [2, 6]. Another study demonstrated a significant relationship between the combination of TS and DPD levels and RFS rates in grade 1 and 2 non-muscle-invasive bladder cancer (Ta, T1). Specifically, patients with both low TS and high DPD activities had a significantly greater RFS rate than patients with either higher TS or lower DPD activities [4]. However, these previous reports did not assess these relationships among patients treated with BCG for T1G3 bladder cancer.

Our results demonstrate that a high DPD level is a significant prognostic factor among patients treated with BCG for T1G3 bladder cancer. Therefore, our results suggest that a DPD inhibitor could potentially be a key agent for successfully treating cases of T1G3 bladder cancer that are resistant to BCG. Previous studies indicated that 5-FU might be a key agent because TS, a target enzyme of 5-FU, was significantly associated with tumor stage, grade, and prognosis in UC [1, 6, 23]. Based on the results of previous studies and the present study, 5-FU based agents with a strong DPD inhibitor such as UFT and S-1 might be a novel treatment for T1G3 bladder cancer patients treated with BCG. Therefore, clinical trials should be performed in the future.

The potential limitations of our study are related to its retrospective design and the relatively small sample size. As a result of the small sample size, we were unable to perform multivariate analyses. Future studies could explore the details of the mechanism by which higher DPD levels influence resistance to BCG therapy.

## Conclusion

In conclusion, the mRNA level of DPD is significantly associated with tumor recurrence as well as stage progression in patients with T1G3 bladder cancer who receive adjuvant BCG therapy. The DPD expression level in tumor tissue might be a strong bio-marker in T1G3 cancer patients receiving BCG therapy.

## References

[1] Mizutani Y, Wada H, Ogawa O, Yoshida O, Fukushima M, Nonomura N, Miki T (2001). Prognostic significance of thymidylate synthase activity in bladder carcinoma. Cancer.

[2] Mizutani Y, Wada H, Fukushima M, Yoshida O, Ukimura O, Kawauchi A, Miki T (2001). The significance of dihydropyrimidine dehydrogenase (DPD) activity in bladder cancer. Eur J Cancer.

[3] Mizutani Y, Wada H, Fukushima M, Yoshida O, Nakanishi H, Li YN, Miki T (2004). Prognostic significance of orotate phosphoribosyltransferase activity in bladder carcinoma. Cancer.

[4] Mizutani Y, Wada H, Yoshida O, Fukushima M, Bonavida B, Kawauchi A, Miki T (2002). Prognostic significance of a combination of thymidylate synthase and dihydropyrimidine dehydrogenase activities in grades 1 and 2 superficial bladder cancer. Oncol Rep.

[5] Kubota Y, Hosaka M, Fukushima S, Kondo I (1993). Prophylactic oral UFT therapy for superficial bladder cancer. Cancer.

[6] Ide H, Kikuchi E, Hasegawa M, Kozakai N, Kosaka T, Miyajima A, Oya M (2012). Prognostic significance of 5-fluorouracil metabolism-relating enzymes and enhanced chemosensitivity to 5-fluorouracil by 5-chloro 2,4-dihydroxy-pyridine in urothelial carcinoma. BMC Cancer.

[7] Sanchez-Carbayo M, Cordon-Cardo C (2007). Molecular alterations associated with bladder cancer progression. Semin Oncol.

[8] Azuma H, Inamoto T, Takahara K, Ibuki N, Nomi H, Yamamoto K, Narumi Y, Ubai T (2012). Neoadjuvant and adjuvant chemotherapy for locally advanced bladder carcinoma: development of novel bladder preservation approach, Osaka Medical College regimen. Int J Urol.

[9] Koga F, Kihara K (2012). Selective bladder preservation with curative intent for muscle-invasive bladder cancer: a contemporary review. Int J Urol.

[10] Simone G, Papalia R, Ferriero M, Guaglianone S, Castelli E, Collura D, Muto G, Gallucci M (2013). Stage-specific impact of extended versus standard pelvic lymph node dissection in radical cystectomy. Int J Urol.

[11] Lamm DL (2000). Preventing progression and improving survival with BCG maintenance. Eur Urol.

[12] Patard JJ, Rodriguez A, Leray E, Rioux-Leclercq N, Guille F, Lobel B (2002). Intravesical Bacillus Calmette-Guerin treatment improves patient survival in T1G3 bladder tumours. Eur Urol.

[13] Thalmann GN, Markwalder R, Shahin O, Burkhard FC, Hochreiter WW, Studer UE (2004). Primary T1G3 bladder cancer: organ preserving approach or immediate cystectomy?. J Urol.

[14] Horikoshi T, Danenberg K, Volkenandt M, Stadlbauer T, Danenberg PV (1993). Quantitative measurement of relative gene expression in human tumors. Methods Mol Biol.

[15] Ichikawa W, Takahashi T, Suto K, Nihei Z, Shirota Y, Shimizu M, Sasaki Y, Hirayama R (2004). Thymidylate synthase and dihydropyrimidine dehydrogenase gene expression in relation to differentiation of gastric cancer. Int J Cancer.

[16] Emmert-Buck MR, Bonner RF, Smith PD, Chuaqui RF, Zhuang Z, Goldstein SR, Weiss RA, Liotta LA (1996). Laser capture microdissection. Science.

[17] Epstein JI, Amin MB, Reuter VR, Mostofi FK (1998). The World Health Organization/International Society of Urological Pathology consensus classification of urothelial (transitional cell) neoplasms of the urinary bladder. Bladder Consensus Conference Committee. Am J Surg Pathol.

[18] Bonner RF, Emmert-Buck M, Cole K, Pohida T, Chuaqui R, Goldstein S, Liotta LA (1997). Laser capture microdissection: molecular analysis of tissue. Science.

[19] Lord RV, Salonga D, Danenberg KD, Peters JH, DeMeester TR, Park JM, Johansson J, Skinner KA, Chandrasoma P, DeMeester SR, Bremner CG, Tsai PI, Danenberg PV (2000). Telomerase reverse transcriptase expression is increased early in the Barrett's metaplasia, dysplasia, adenocarcinoma sequence. J Gastrointest Surg.

[20] Atzpodien J, Royston P, Wandert T, Reitz M (2003). Metastatic renal carcinoma comprehensive prognostic system. Br J Cancer.

[21] Lebret T, Becette V, Barbagelatta M, Herve JM, Gaudez F, Barre P, Lugagne PM, Botto H (1998). Correlation between p53 over expression and response to bacillus Calmette-Guerin therapy in a high risk select population of patients with T1G3 bladder cancer. J Urol.

[22] Alvarez-Mugica M, Cebrian V, Fernandez-Gomez JM, Fresno F, Escaf S, Sanchez-Carbayo M (2010). Myopodin methylation is associated with clinical outcome in patients with T1G3 bladder cancer. J Urol.

[23] Li Y, Li X, Dai H, Sun X, Li J, Yang F, Gu H, Yang Y, Jin Z, Chu Y, Jin X, Kakehi Y, Wu X (2009). Thymidylate synthase was associated with patient prognosis and the response to adjuvant therapy in bladder cancer. BJU Int.

